# Gestational pemphigoid

**DOI:** 10.1186/s13023-014-0136-2

**Published:** 2014-09-02

**Authors:** Laura Huilaja, Kaarin Mäkikallio, Kaisa Tasanen

**Affiliations:** Department of Dermatology, Medical Research Center, University of Oulu, Oulu University Hospital, Oulu, Finland; Department of Obstetrics and Gynecology, University of Oulu, Oulu University Hospital, Oulu, Finland

## Abstract

Gestational pemphigoid (pemphigoid gestationis, PG) is a rare autoimmune skin disorder occurring characteristically during pregnancy. Autoantibodies against placental BP180 (also known as BPAG2 or collagen XVII) cause damage to the skin basement membrane, resulting in severe itching and blistering rash over the body and the extremities. The diagnosis of PG is confirmed by immunofluorescence analysis of a skin biopsy, while serum levels of pemphigoid antigen BP180 antibody can be used to assess disease activity. PG with mild symptoms can be treated with topical corticosteroids, while oral corticosteroids are the mainstay in treatment of severe PG. PG usually flares up at the time of delivery, and resolves spontaneously shortly after. However, relapses in subsequent pregnancies are common. As PG has been linked to the risk of prematurity and fetal growth restriction, prenatal monitoring jointly by a dermatologist and an obstetrician is recommended. Mothers should also be informed of the potential risk of re-activation of the disease in subsequent pregnancies and during hormonal contraception.

## Introduction

Gestational pemphigoid (pemphigoid gestationis, PG) is a rare autoimmune skin disorder that occurs during pregnancy. PG belongs to the pemphigoid group of autoimmune skin diseases that cause blistering of the skin and mucosal membranes [1]. The most common form is bullous pemphigoid (BP); other major forms include mucous membrane pemphigoid and linear IgA disease. In pemphigoid diseases, autoantibodies target hemidesmosomal proteins that maintain adhesion between basal keratinocytes and the basement membrane, thereby breaking cell-matrix adhesion and typically causing subepidermal blisters. These proteins include bullous pemphigoid antigen 180 (BP180, i.e., BPAG1 or collagen XVII) and BP230 (i.e., BPAG1-e). The IgG autoantibodies to BP180 are pathogenic but the role of autoantibodies against BP230 in blister formation is unclear [1].

PG was previously called herpes gestationis, but this misnomer should be withdrawn, since there is no true connection to herpetic diseases [2]. Studies looking for the epidemiology of PG are rare. Population-based studies have reported an annual incidence ranging between 0.5 and 2.0 cases per 1 million people in France, Kuwait and Germany [3–5]. In a retrospective study, PG was found in 4.2% of 505 pregnant patients evaluated in university-based dermatologic pregnancy clinics [6]. Based on the current epidemiological data PG is estimated to occur in one out of about 40,000-50,000 pregnancies [7] with no difference in racial distribution [8,9]. Single cases have been described in association with molar pregnancies [10] and trophoblastic tumors [11].

### Clinical features

**Figure 1:**
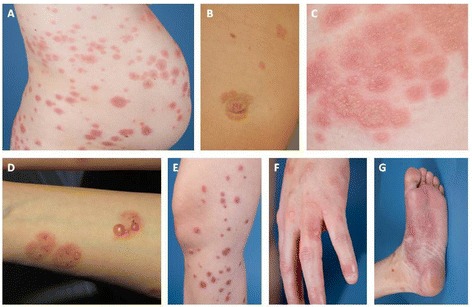
**Skin findings of gestational pemphigoid (PG).** Urticarial papules and plaques usually appearing first on abdominal area **(A)**. Minor umbilical lesions of PG **(B)**. Vesicles **(C)** and bullae **(D)** following urticarial plaques. PG lesions on extremities **(E-G)**.

PG may appear at any time during pregnancy or puerperium, but the most common time of symptom onset is during the second and third trimester. Intense abdominal itching usually begins around the navel, with varied red papules, urticarial plaques or annular target lesions (erythema multiforme –like) appearing in the itchy areas, followed by blistering after a few weeks (Figure 1). Bullous lesions vary from small vesicles to large blisters with a thick roof; however, some PG patients have no blisters at all (Figure 1). Typically, the skin symptoms first appear in the abdominal area, but according to an American study (n = 10) it is also common for cutaneous manifestations to appear first in the extremities [12]. In a Finnish study (n = 12) the symptoms started in the abdominal area in all patients, and 92% developed blisters as the disease progressed [13]. Facial and mucosal lesions are uncommon [12,14], but in some reports severe mucosal lesions were associated with more persistent disease [15].

The symptoms of PG usually alleviate a few weeks before delivery, but the disease is re-activated in 75% of the patients at the time of delivery. The remitting, relapsing course of the disease has been thought to be associated with progestin, which has immunosuppressive properties, and with changes in progestin levels: an increase in late pregnancy followed by a sharp fall during delivery [7,16]. According to a large PG study (n = 87), the average duration of symptoms is 16 weeks and the majority of mothers are symptom-free 6 months after the delivery, the duration of postnatal manifestations varying between 2 weeks and 12 years [16].

### Etiopathology

The pathogenesis of PG remains unknown. The presence of MHC II-class HLA-antigens DR3 and DR4 or their combination has been shown to be clearly more common in women with PG compared to normal population [17]. Placental and fetal tissues contain paternal tissue antigens that are foreign to the maternal immune system. However, the maternal immune system does not normally react against these foreign antigens. In patients with PG, MHC II-class molecules that are normally not present in the placenta have been detected in trophoblastic placental cells and amniochorionic stroma cells. As a result of partial breakdown of the syncytiotrophoblast cell layer of placental anchor villi, MHC II molecules are thought to get in contact with the maternal immune system, causing a (semi) allogeneic immune reaction against the BP180 molecule [18–20].

BP180 (also known as BPAG1 or collagen XVII) is a key structural protein of hemidesmosomes linking the epidermis and dermis. It consists of a short intracellular domain and a large extracellular domain [21]. Besides the skin basement membrane zone, BP180 is found in the placental tissue and fetal membranes. Placental BP180 is detectable in cytotrophoblastic cells as early as from the first trimester [22]. In PG, antibodies are mainly directed against the same BP180 epitopes as in bullous pemphigoid [23,24]: most commonly against the epitopes found in NC16A, the largest non-collagenous domain of BP180, but antibodies against intracellular BP180 domains and other extracellular domains of BP180 have also been observed [25]. In addition, antibodies against another structural basement membrane protein, BP230, have been detected in about 10% of patients with PG, but this is considered to be secondary and clinically insignificant [7,26]. The cross-reaction between placental antibodies and skin BP180 causes the typical skin symptoms of PG [7,27].

To the best of our knowledge, PG-related animal models do not exist. In BP, the pathogenicity of autoantibodies directed against the NC16A domain has been confirmed in several mouse models [1,28]. Severe blistering and high mortality prevents the use of experimental BP-mice for breeding to imitate a PG-like condition. The transfer of autoantibodies from mother with PG to newborn is to some extent simulated in a gene-targeted mice model in which the maternal transfer of NC16A antibodies results in blistering in the neonatal BP180-humanized mice [29].

### Diagnosis

**Figure 2:**
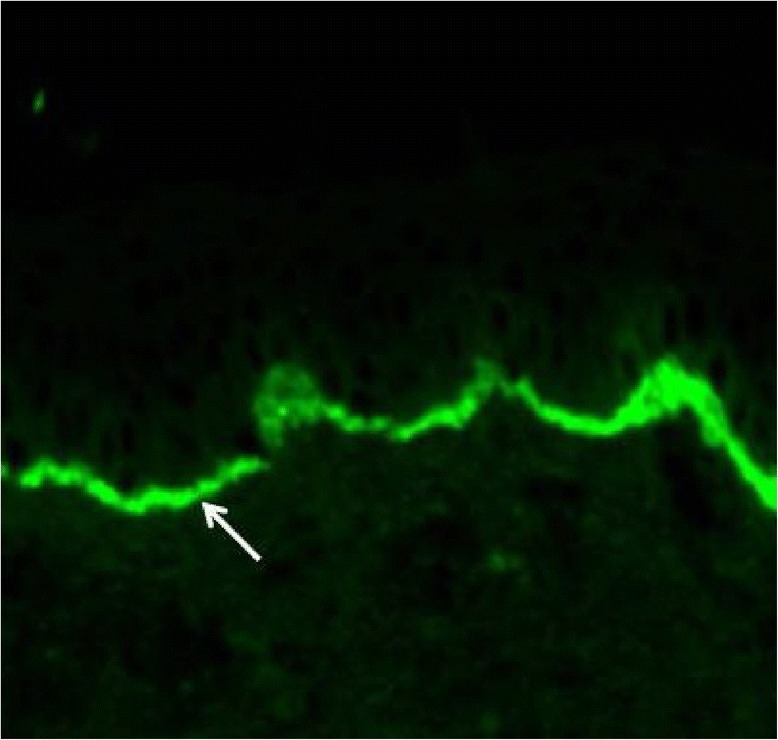
**Linear complement 3 (C3) (arrow) fluorescence in immunofluorescence analysis of perilesional skin biopsy is diagnostic for gestational pemphigoid.** Primary magnification 200 × .

The diagnosis of PG is preferably made by a dermatologist, but all physicians treating pregnant women, i.e., general practitioners and obstetricians, should be able to consider PG. A biopsy for histopathology is not needed; the diagnosis is based on clinical picture, direct immunofluorescence microscopy and serology [1,30,31]. Direct immunofluorescence examination of a snap-frozen perilesional skin biopsy reveals the linear accumulation of complement C3 in the basement membrane zone at the interface of the epidermis and dermis (Figure 2). Linear IgG positivity is also detected in about 25-50% of the samples, but it is not a criterion for the diagnosis [27,32]. If PG is suspected, measurement of serum BP180 antibody level is recommended, as it correlates with the degree of disease severity and facilitates assessment of treatment response [33,34]. Since the BP180 NC16A ELISA is sensitive and specific to PG, it has even been proposed as a PG screening test or to be sufficient for the PG diagnosis in conjunction with typical clinical symptoms [33–35]. Serum autoantibodies can also be detected with traditional indirect immunofluorescence microscopy or the complement binding test on salt-split skin [1,30,31].

### Differential diagnosis

Since PG is an extremely rare condition, other dermatologic reasons for itchy cutaneous eruptions (Table 1) occurring during pregnancy should be ruled out. Pregnancy may influence the clinical picture of common skin diseases that either precede pregnancy or coincide with it by chance. Especially PG with atypical symptoms such as non-intense pruritus, mild erythematous papules and plaques or eczematous lesions represents a true challenge for clinical diagnostics.

**Table 1:** **Differential diagnostics of pregnancy associated pruritic dermatoses**

	**Atopic eruption of pregnancy**	**Polymorphic eruption of pregnancy**	**Intrahepatic cholestasis of pregnancy**	**Gestational pemphigoid**
**Estimated incidence**	Most common pregnancy associated dermatose. 1:5–1:20	1:160	1:50–1:5000	1:40000–1:50000
**High-risk groups**		Primigravidity		Multiparity
		Obesity		
		Multiple pregnancy		
**Skin manifestations**	Pruritus	Pruritus	(Nocturnal) pruritus	Pruritus
	Eczematous lesions	Urticarial papules and plaques	Secondary skin lesions due to scratcing	Papules
				Urticarial plaques
				Target lesions
				Blisters, vesicles
	Papules	Sparing of the umbilical region	Jaundice	
**Localization of skin manifestations**	Trunk	Lower abdomen	Extremities (palms and soles)	Abdomen, umbilicus
				Extremities
	Extensors of the extremities	Striae		
		Thighs		
		Body		
**Studies**	S-IgE levels may be elevated	Negative DIF	Elevated total serum bile acid levels	Linear C3 (and IgG) positivity in DIF. BP180 ELISA
**Symptom onset** (trimester of pregnancy)	I-II	III	III	II-III
**Parturition/Lactation**	Symptom resolution	Symptom resolution	Symptom resolution	Flare-up in connection to delivery
**Pregnancy complications**	No fetal risks	No fetal risks	Stillbirth	Prematurity
				Fetal growth restriction
**Newborn**	No harm to newborn	No harm to newborn	No harm to newborn	Possibility for transient skin blistering
**Recurrence**	No elevated risk for recurrence	No elevated risk for recurrence	Elevated risk for recurrence	Recurrence is usual.
				Activation of symptoms is possible during menstruation and hormonal contraceptive use

S-IgE: serum immunoglobulin E; DIF: direct immunofluorescence microscopy; BP180-ELISA: bullous pemphigoid 180 ELISA.

The most important differential diagnosis alternatives for PG are the other specific dermatoses of pregnancy which include atopic eruption of pregnancy (AEP), polymorphic eruption of pregnancy (PEP) and intrahepatic cholestasis of pregnancy (ICP) [6,36–40]. AEP is the most common pregnancy-specific skin disease, which typically appears in the first and second trimesters [40]. About 20% of the patients with AEP have a pre-existing atopic dermatitis with a typical clinical picture, whereas the remaining 80% present widespread eczematous changes or papular lesions and have no previous history of atopic eczema or have been symptomless since childhood [31]. The greatest differential diagnostic challenge of PG is PEP, previously known as Pruritic Urticarial Papules and Plaques of Pregnancy (PUPPP), with intensely pruritic urticarial papules and plaques during the last trimester. Despite rather similar clinical features, negative immunofluorescence analysis of perilesional skin biopsy in PEP differentiates it explicitly from PG [38,39]. Similar to PG, PEP symptoms usually start on the abdomen, but PEP lesions typically spare the umbilical region. ICP, which is associated with significant fetal risks, can present in the last trimester with pruritus, and thus it should be considered in differential diagnosis of PG [40]. Patients with ICP do not have primary skin lesions, but due to severe pruritus and scratching may develop secondary excoriations or even prurigo nodularis-like changes, usually on the extremities [31].

### Management

Due to the rarity of PG no randomized studies have been published and treatment recommendations are based on clinical experience and studies from treatment of other skin diseases. PG symptoms can be quite debilitating, but the condition does not constitute a direct health risk to the mother. When choosing a treatment, the benefit of the medication to the mother is critically weighed up against possible risks to the fetus. The aim of the treatment is to suppress the excessive itching and to prevent formation of new blisters [41].

According to current recommendations PG patients with mild symptoms (about 19% of the patients) should be treated with potent or very potent topical corticosteroids (for example betamethasone valerate or clobetasol propionate) [1,30]. Controlled studies with BP patients have shown that topical treatment with highly potent corticosteroid is as effective and safe as oral prednisolone 0.5 mg/kg/day [42]. During pregnancy, mild or moderate topical corticosteroids are preferred to potent or very potent ones because of the risk of fetal growth restriction associated with the latter [43]. When necessary, potent or very potent topical corticosteroids can be used for the therapy of PG for as short duration as possible, since their potential for fetotoxicity is less than that of systemic corticosteroids [43–45].

The combination of oral antihistamines with topical corticosteroids, most commonly cetirizine, is usually employed to relieve the itching, despite the fact that clinical efficacy studies in PG are lacking [1,16,27,30]. In general, second-generation H1-antihistamines are currently preferred to first-generation antihistamines based on the potential serious anticholinergic and central nervous system side effects of old sedating antihistamines and the longer-lasting antipruritic effects of the modern antihistamines [46]. First-generation antihistamines have no definitive increased teratogenic risk, and the second-generation antihistamines cetirizine, levocetirizine and loratadine are also recommended for use in pregnancy [44,46].

Corticosteroid treatment has become the standard of care for first-line systemic therapy of severe PG thanks to its treatment response and tolerable safety profile. Most of prednisolone is inactivated by placental dehydrogenase enzyme (11-hydroxysteroid dehydrogenase-2) before reaching the fetal circulation. As fluorinated corticosteroids (betamethasone and dexamethasone) are not metabolized by placental dehydrogenase enzyme, prednisolone is considered the primary treatment alternative. [1,30,47]. The initial dose of prednisolone is usually 0.25-0.5 mg/kg/day, and the response is usually good. If formation of blisters does not decrease within a few days, the dose is increased until no new blisters appear. The cortisone dose is gradually decreased about 1–2 weeks after the symptoms have been brought under control, and discontinued altogether if possible.

The side effects of long-term systemic corticosteroid treatment are well-known. Previous studies have demonstrated that in the treatment of BP the use of oral prednisolone is associated with more frequent severe adverse events and increased mortality compared to topical corticosteroids [1,30,42]. However, BP patients are much older and have more severe comorbidities than PG patients. In addition, the duration of prednisolone treatment is shorter and the dosage is smaller in PG than in BP, which further decreases the risk of side effects. During pregnancy, the use of prednisolone in the first trimester causes an increased risk of malformations, especially orofacial clefts [44]. In the last trimester prednisolone may result in intrauterine growth retardation, gestational diabetes, eclampsia and premature delivery [44].

Plasmapheresis [48], immunoadsorption [49,50] and intravenous immunoglobulin G-infusion [51–54], which are not contraindicated during pregnancy, have in some cases been used to treat PG even prior to the delivery. Removal of antibodies with immunoadsorption gives quick symptom relief especially in PG cases with severe postnatal symptoms, as there is no placenta to maintain an autoimmune reaction [50]. Prenatal treatment with cyclosporine combined to prednisolone has been reported in two cases with good treatment response [13,55], and in one case cyclosporine was used after intravenous immunoglobulin in persistent postnatal PG [56]. Case reports on the use of tetracycline, cyclophosphamide, azathioprine, dapsone and rituximab to treat PG with persisting postnatal symptoms have been published, but these agents are avoided prenatally due to potential short- and long-term fetal effects. [7,41].

PG lesions usually disappear 12–16 weeks after the delivery, with no scarring, and postnatal oral cortisone treatment can normally be discontinued fairly soon. However, sometimes treatment has to be resumed as the disease flares up again [16,27]. When systemic cortisone is given at the average doses used in the treatment of PG, it does not prevent breastfeeding, and breastfeeding has been shown to reduce the symptoms of PG [17,7,12].

### Fetus and the newborn

The risk of preterm birth and fetal growth restriction is greater in PG pregnancies compared to normal population [57–60]. The pregnancy risks of PG are thought to be associated with mild placental failure caused by BP180 antibodies [13,27,60]. In addition to accumulation of C3 complement and IgG, mild villitis and collections of immature fibrotic villi have been observed in histologic examinations of PG placentas [22]. Antibody concentrations do not as such correlate with the occurrence of pregnancy complications, and no association has been demonstrated between cortisone treatment and PG pregnancy complications [60]. No follow-up guidelines for pregnancies complicated by PG have been published, most likely due to the rarity of the condition.

In the largest data set on PG pregnancies (n = 87) published in 1999 the rate of miscarriages was comparable to the risk in normal population (15%), with the majority of miscarriages occurring in the first trimester [16]. However, in a more recent British-Taiwanese study with 70 patients late miscarriages and fetal deaths were observed in as many as 6% of the patients [60].

About 16-34% of PG patients are estimated to give birth prematurely [13,58–60]. Premature delivery is more likely if PG begins in the 1st or 2nd trimester or if the skin symptoms include blistering [60]. In a Finnish PG study, 25% of the deliveries were premature (the corresponding rate in the Finnish population during time of study was around 5%) [13,61]. The proportion of premature deliveries among pregnant women with PG was similar to that in previously published studies, even though all patients, with one exception, had blistering PG. All premature births occurred after the 35th gestational week, and PG had no effect on neonatal mortality [13]. Vaginal ultrasound is considered the gold standard in charting cervical dilation in women at risk of preterm delivery [62]. Although preterm delivery is difficult to predict, we recommend obstetric follow-up with vaginal ultrasound due to the increased risk of preterm delivery.

In the British-Taiwanese study with 70 patients, fetal growth restriction was observed in 34% [60], the likelihood of its occurrence correlating with early onset of PG. In a Finnish study, only one mother developed pre-eclampsia combined with fetal growth restriction, which is in line with the general prevalence in Finnish population. However, 50% of the patients in our study had an abnormal placental weight/birth weight ratio [13]. The blood flow profile of the umbilical artery is used in clinical practice to diagnose placental failure [63]. In a PG case report where pregnancy was complicated by pre-eclampsia and fetal growth restriction, abnormal end-diastolic blood flow was reported in the umbilical artery [64]. Among 12 Finnish PG patients increased umbilical artery pulsatility was detected only in one pregnancy with pre-eclampsia and fetal growth restriction; all other PG pregnancies showed normal umbilical artery blood flow findings and biophysical scores [13], suggesting that clinically significant placental failure is rare in PG.

There is only little information available on the effect of PG on the newborn infant. No congenital abnormalities have been linked to PG [58,60]. According to the data from 12 Finnish PG patients, birth weight, umbilical artery pH, Apgar scores and neonatal morbidity did not differ from normal population [13]. The IgG antibodies of PG pass through the placenta, but PG blisters develop in only about 3% of newborn infants [14,16,57]. Skin symptoms in newborns usually resolve quickly without treatment as antibody levels decrease. According to a Japanese case report, antibody levels in newborn infants are comparable to those in mothers; the levels in both are reduced by half in about 15 days. Since the clinical status of the newborn often improves rapidly, it seems that other factors besides autoantibodies contribute to the formation of blisters in newborns [65]. If PG in the mother was treated with large doses of cortisone, the pediatrician should be informed of the possibility of neonatal adrenal insufficiency. There are no data on the long-term prognosis of children of PG mothers.

### Prognosis

Recurrence of PG in subsequent pregnancies is likely, and symptoms are usually more severe, with earlier onset. In patients with an earlier PG episode the likelihood of pregnancy with no symptoms is estimated to be 5-8%, but the reason for the lack of symptoms is unknown. [16,17]. In a Finnish study, PG recurred in two cases, while 67% of the subsequent pregnancies were symptom-free (n = 4/6). The large proportion of symptom-free pregnancies is most likely due to the small number of patients, but ethnic factors may also play a role [13]. Perfect HLA-DR match between the mother and the fetus may explain some of the cases, but pregnancy may be free from PG symptoms even in the absence of identical HLA-DR type [16,17] and even if the symptoms of PG have been persistent [66].

At the postpartum examination, mothers with PG should be reminded of the possibility of relapse during menstruation and/or in connection with hormonal contraceptive use. Susceptibility for recurrence may persist for years [16,37]. In a large British study, about 11% of the patients experienced a relapse during oral contraceptive use, but in smaller studies the incidence has been as high as 50% [14,16,17]. The low relapse rate in the British study was thought to be associated with the infrequent use of oral contraceptives after PG pregnancy [16]. Women who have had PG have also been described to be more susceptible to other autoimmune diseases; the prevalence of Graves’ disease increases in particular, from 0.4% in normal population to as high as 10.6% [16]. Proneness to Hashimoto’s thyroiditis, autoimmune thrombocytopenia and pernicious anemia has also been reported to be increased [16,67].

## Conclusion

Gestational pemphigoid is a rare skin disorder in pregnancy. The severe itching and blistering caused by the disease can be quite debilitating. The diagnosis of PG is made in a specialized care setting at a dermatology department. Since PG is associated with a risk of prematurity and fetal growth restriction, pregnancy monitoring by an obstetrician is recommended. Mothers with PG should be informed of the natural course of the disease, good fetal prognosis, the possibility of relapses after delivery, and the risk of relapses in subsequent pregnancies and with hormonal contraception.

## Abbreviations

PG: Pemphigoid gestationis, gestational pemphigoid
BP: Bullous pemphigoid
BP180: Bullous pemphigoid antigen 180
BP230: Bullous pemphigoid antigen 230
MHC: Major histocompatibility complex
HLA: Human leucocyte antigen
IgG: Immunoglobulin G
AEP: Atopic eruption of pregnancy
PEP: Polymorphic eruption of pregnancy
ICP: Intrahepatic cholestasis of pregnancy
